# Earl Stanhope's Account of the Mental Malady of George III

**Published:** 1862-04

**Authors:** 


### EARL STANHOPE'S ACCOUNT OF THE MENTAL MALADY OF GEORGE III.

The following highly graphic account of the mental disorder which afflicted George III. in 1788, 1801, and 1804, is from the recently published Life of William Pitt, by Earl Stanhope:—

1788.—"The constitution of George III. was by nature hardy and robust; but with a constant tendency to corpulence. To counteract this the King had from an early period adopted a system of abstemious diet and of active exercise. While his meals were of the simplest and plainest kind, the equerries in attendance upon him might often complain of the great distances which he rode in hunting, or of his walks of three hours before breakfast. This system carried to excess, combined with never-failing and anxious attention to affairs of state, was the cause of the mental malady in 1788. Such, at least, was the opinion of the case expressed by Dr. Willis, the ablest by far of his physicians, when examined by the Committees of the House of Lords and House of Commons.

"Early in the summer of 1788 the King's health suffered from repeated bilious attacks. In a letter to Mr. Pitt he says of himself that he is certainly 'a cup too low.' His physicians prescribed the waters of Cheltenham, and on the 12th of July, the day after the prorogation, he set out with the Queen for that place. A sojourn of several weeks failed, however, to yield him the expected benefit. When he returned, first to Kew, and afterwards to Windsor, he seemed weaker in body than before. His attendants were surprised and grieved at seeing him, so lately the most athletic of pedestrians, require the support of a stick. 'I could not,' he said, 'get on without it; my strength seems diminishing hourly.' 'My dear Effy'—thus he accosted one of the Queen's ladies, the Dowager Countess of Effingham—'you see me at once an old man!'

"Yet still, in some points at least, the King's active habits were maintained. Mr. Rose reports that 'Mr. Pitt saw him at Kew, and was with him three hours and forty minutes, both on their legs the whole time.' And this brings us to a peculiarity in the reign of George III. It was the invariable or almost invariable custom of that monarch to confer with his ministers standing, neither himself to sit down nor ask them to be seated. This rule, so highly inconvenient to both parties, was no doubt derived from some of the Continental Courts.

"At this period of October, 1788, the only physician in attendance on the King was Sir George Baker. He states in his evidence before the subsequent committees, that the first time when he conceived any suspicion of a mental malady in the King, was in the evening of the 22nd of October. Next morning the unfavourable symptoms which led to that suspicion had wholly disappeared.

"On the 24th, however, the King made an effort beyond his strength, in going to hold a levee at St. James's. He made that effort, as he wrote to Mr. Pitt, 'to stop further lies, and any fall of the Stocks.' But at the levee his manner and conversation were such as to cause the most painful uneasiness in several at least of those to whom he spoke. Mr. Pitt, in particular, could not entirely suppress his emotion when he attended the King in his closet after the levee, which His Majesty observed and noticed with kindness in writing next day to his minister from Kew. Probably conscious himself, at least in some degree, of his coming malady, he directed Mr. Pitt, in the same letter, not to allow any political papers to be sent to him before the next ensuing levee.

"On the 25th the King removed to Windsor Castle. His state appears to have fluctuated from day to day; but there was no lasting improvement in his health. His letters to Mr. Pitt, which I shall give at length in my appendix, bear no tokens of an incoherent mind. They merely manifest some reluctance and anxiety as to the measures which Pitt desired to pursue with regard to the Northern Powers. The last letter of the King before his malady is dated on the 3rd of November. In this His Majesty states that he can now sign warrants in any number without inconvenience. He adds, that he attempts reading the despatches daily, but as yet without success.

"Of the King's real condition at this time, by far the best, and indeed, so far as published, the only good account, is to be found in the private journal of Miss Frances Burney, the accomplished author of 'Evelina.' That lady was now a member of the royal household, and in daily attendance on the Queen as, under Mrs. Schwellenberg, Deputy Keeper of the Robes. Dull and trifling as the earlier volumes of her 'Diary,' I must confess, appear to me, the entries in it now become of lively interest and of sterling value, and are marked by not merely dutiful, but warm and affectionate attachment to her Royal Mistress.

"By some extracts from her journal my narrative may be best continued:—

"'*Sunday, November* 3.—We are all here in a most uneasy state. The King is better and worse so frequently, and changes so daily backward and forward, that everything is to be apprehended if his nerves are not someway quieted. I dreadfully fear he is on the eve of some severe fever. The Queen is almost overpowered with some secret terror. I am affected beyond all expression in her presence to see what struggles she makes to support serenity. To-day she gave up the conflict when I was alone with her, and burst into a violent fit of tears. It was very, very terrible to see!

"*Wednesday, November* 5.—I found my poor royal mistress in the morning sad and sadder still; something horrible seemed impending; and I saw her whole resource was in religion. We had talked lately much upon solemn subjects; and she appeared already preparing herself to be resigned for whatever might happen.

"At noon the King went out in his chaise with the Princess Royal for an airing. I looked from my window to see him: he was all smiling benignity, but gave so many orders to the postilions, and got in and out of the carriage twice with such agitation, that again my fear of a great fever hanging over him grew more and more powerful. Alas! how little did I imagine I should see him no more for so long—so black a period!

"When I went to my poor Queen, I found her spirits still worse and worse . . . . The Princess Royal soon returned. She came in cheerfully, and gave in German, a history of the airing, and one that seemed comforting.

"Soon after suddenly arrived the Prince of Wales. He came into the room. He had just quitted Brighthelmstone. Something passing within seemed to render this meeting awfully distant on both sides. She asked if he should not return to Brighthelmstone. He answered, 'Yes; the next day.' He desired to speak with her; they retired together."

"This day, the 5th of November, of which Miss Burney has thus described the earlier portion, proved to be the first of the King's disorder, when its real nature could be no longer mistaken or concealed. For that afternoon the King, at dinner with the Royal Family, broke forth into positive delirium, and the Queen was so overpowered as to fall into violent hysterics.

"Next morning, the 6th, when Miss Burney rose, she found that the equerries and gentlemen in attendance had sat up next his chamber door all night, and there were likewise all the pages dispersed in the passages and ante-rooms; 'and oh,' she adds, 'what horror in every face I met!'

"Besides Sir George Baker, who continued in close attendance, a physician of the highest eminence—Dr. Warren—had been sent for by express. When, however, he came, the King positively refused to see him. 'This was terrible,' writes Miss Burney. 'But the King was never so despotic; no one dared oppose him. He would not listen to a word, though when unopposed he was still all gentleness and benignity to every one around him . . . . He kept talking unceasingly; although his voice was so lost in hoarseness and weakness it was rendered almost inarticulate.'

"Expresses had of course gone up also to Mr. Pitt. His grief may be easily imagined; but his anxiety was not less than his grief. He saw at once the difficulties that rose before him in the event that the King's reason should continue clouded, and yet his life be spared. In such a case there were strong grounds for imposing some restrictions on a Regency. Yet how could such restrictions be imposed unless by Act of Parliament? and how could any Act of Parliament be passed without a King to give his assent? Thus, in one sense, a limited Regency seemed requisite, while in another sense it seemed impossible.

"Pitt, however, applied himself at once to all the measures in his power. This same afternoon he sent expresses to summon the cabinet ministers who were absent from town. Here is his letter to the Marquis of Stafford, Lord Privy Seal.

Grosvenor Square, *Nov.* 6, 1788, 6 P.M.

"My dear Lord,—I write from Lord Carmarthen's, having just heard an account from Windsor, by which I learn that the King's disorder, which has for some days given us much uneasiness, has within a few hours taken so serious a turn, that I think myself obliged to lose no time in apprising your lordship of it.

"The accounts are sent under considerable alarm, and therefore do not state the symptoms very precisely; but from what I learn, there is too much reason to fear that they proceed from a fever, which has settled on the brain, and which may produce immediate danger to his Majesty's life. You will easily conceive the pain

I suffer in being obliged to send your lordship this intelligence; but as you may possibly think it right, under such circumstances, to be on the spot as soon as possible, I thought no time should be lost in letting you know the situation.

"I am, with great regard, &c.,

"W. Pitt."

"On the same day Pitt also wrote to the Bishop of Lincoln, at Buckden Palace, and here is the extract from his letter which the Bishop gives:—

"The effect most to be dreaded is on the understanding. If this lasts beyond a certain time, it will produce the most difficult and delicate crisis imaginable, in making provision for the Government to go on. It must, however, be yet some weeks before that can require decision; but the interval will be a truly anxious one. You shall hear again soon; but if in the course of a few days you could spare the time to come to town, I should be very glad to talk with you, as there will be a thousand particulars you must wish to know, which I cannot write. I shall not stir from hence, except for going to inquire at Windsor."

The Bishop adds:—

"I went to town immediately, and late at night found Mr. Pitt expecting a messenger every moment with the account of the King's death; but the intelligence, which did not arrive till two in the morning, proved more favourable."

"During the night which followed there were many anxious watchers in the apartment next to the Royal sufferer's. The Prince of Wales, the Duke of York, the physicians, and the gentlemen of the royal household, sat on chairs and lay on sofas round the room. All were in dead silence, and amidst the partial darkness the two princes were still to be distinguished by their stars.

"Next day, 7th of November, towards seven in the morning, when the Queen was already dressed, but Miss Burney was still attending her, the Prince of Wales came hastily into Her Majesty's chamber, and then, in Miss Burney's presence, gave 'a very energetic history' of the preceding night. The King had risen some hours before daylight, and insisted on walking into the next apartment. There he was utterly amazed at finding, instead of the mere solitude which he expected, the large assemblage of his family and household. With some haste, he demanded what they all did there. Sir George Baker was exhorted in whispers by the gentlemen near him, and even, it would seem, by the Prince of Wales, to lead the King back to his chamber; but he had not courage, and he seems, indeed, to have well deserved the character which the King presently gave him, when His Majesty pinned him in a corner and told him he was only an old woman. No one else dared approach his Majesty, and this most painful scene continued a considerable time. At length the Queen's Vice-Chamberlain, Colonel Stephen Digby, an old servant of their Majesties, resolved to act. He went boldly up, and taking the King by the arm, entreated him to go to bed; but finding entreaties in vain, began to draw his Majesty along, and to say he must go. 'I will not,' cried the King, 'I will not! Who are you?' 'I am Colonel Digby, sir,' he answered, 'and your Majesty has been very good to me, and now I am going to be very good to you; for you must come to bed, sir—it is necessary to your life.' And then, continued the Prince of Wales in his narrative, the King was so surprised that he allowed himself to be drawn along as gently as a child, and thus was he brought back to his chamber.

"Hear, then, was the turning-point. This was the precise moment when ceased the dominion of a sovereign over his subjects, and when began, on the contrary, the dominion of sound minds over an unsound one. Here, then, let history pause. So long as the King continued a public character, it is her right and her duty to record his course; not so to explore the dismal secrets of his enforced and lonely sickroom.

"It may therefore suffice to say, in general terms, that during the next few days the King became greatly worse, both in mind and body. Not only seemed his reason lost, but his life in imminent danger. Then, in those hours of suspense and anguish at Windsor, came to light some further revelations of his growing malady. The Queen had sent for Dr. Warren, soon after his first arrival, and felt it her duty to inform him privately that for some time past she had more than suspected the real situation of the King. The Duke of York had met the King on Monday the 3rd, after his Majesty had been on horseback for some hours, and the King, drawing his son aside, had burst into tears and given utterance to the simple, but most affecting words, 'I wish to God I might die, for I am going mad!'

"The physicians in daily attendance—and within a fortnight their number had been increased to four—were of course guarded and cautious in their expressed opinions. But among the members of the royal household the belief was most strongly prevalent that there was little or no prospect of the King's recovery. The Queen withdrew to her own chamber, and passed the whole day in patient sorrow with her daughters. The entire direction of the household devolved upon the Prince of Wales, and nothing at Windsor was done but by direction of his royal highness.

"So great and awful an affliction, and so deep a responsibility resulting from it, could not fail to impress even the least earnest minds.

"'The Prince was frightened, and was blooded yesterday.' So wrote one of the Grenville cousins who was at Windsor on the 7th.* * * *

"Meanwhile the illness of the King and its real nature could not be kept secret. The tidings of it flew far and wide throughout the country, everywhere exciting the utmost sympathy and sorrow. Then did it become apparent how strong and deeply rooted was in truth at this period the popularity of George the Third, and how thoroughly had passed away from it the clouds of earlier years. By the Queen's direction, Colonel Digby had written to the Archbishop of Canterbury, suggesting that there should be offered up public prayers for the King's recovery. A form of prayer was accordingly framed at Lambeth Palace, and ordered to be used in all the churches, and the manner in which the various congregations through the kingdom joined in this act of worship clearly evinced the sincerity and the strength of their affliction.

"Other manifestations of the same feeling were not all as commendable. Thus the physicians in attendance on the King received every day a number of threatening letters to answer for the safety of their monarch with their lives. On one occasion, Sir George Baker was stopped in his carriage by the mob, and required to give an account of the King's state. Poor Sir George faltered out that it was a bad one; on which there arose a furious cry: 'The more shame for you!'† * * *

"Soon afterwards a case of much difficulty arose at Windsor. There was a strong and just desire to remove his Majesty to Kew. In the first place the distance of Windsor from London was most inconvenient to the physicians in attendance; secondly, and this was the main if not the only reason put forward by themselves, it was most essential that the King should have, as he might have at Kew and could not at Windsor, a private garden, where, whenever his health permitted, he might take exercise without being overlooked or observed. But, on the other hand, the King showed the most extreme repugnance to leave Windsor. It was thought that even in his distracted state the advice of his confidential servants would have weight with him, and the necessity of compulsion be thus avoided. Accordingly, on the 28th, a privy council was held at Windsor, when the physicians were formally examined, all agreeing that the removal of his Majesty to Kew was a point of most pressing importance.

* Vol. i., pp. 383—392. † Vol. i., p. 393.

"For the scene that followed, as for some of the preceding, I adopt the graphic description of Miss Burney:—

'Inexpressible was the alarm of every one lest the King, if he recovered, should bear a lasting resentment against the authors and promoters of this journey. To give it therefore every possible sanction, it was decreed that he should be seen both by the Chancellor and Mr. Pitt. The Chancellor went into his presence with a tremor such as before he had been only accustomed to inspire, and when he came out he was so extremely affected by the state in which he saw his royal master and patron, that the tears ran down his cheeks, and his feet had difficulty to support him. Mr. Pitt was more composed, but expressed his grief with so much respect and attachment, that it added new weight to the universal admiration with which he is here beheld.'

"But whatever may have passed at these most painful interviews, it was found that they had not surmounted the morbid aversion of the King to the change required. 'In what a situation was the house!' exclaims Miss Burney; 'princes, equerries, physicians, pages—all conferring, whispering, plotting, and caballing how to induce the King to set off!' Recourse was now had to a no less painful stratagem. The King had for some time been most earnestly pressing to see the Queen and the Princesses, but this the Physicians had deemed it necessary to refuse him. It was then decided that the royal ladies should proceed early next morning to Kew; that the King should be informed of their departure by the physicians: and that if, as they expected, he should doubt their assertion, he might be suffered to go through the apartments and ascertain the fact for himself. Next a promise was to be made his Majesty that on rejoining the members of his family at Kew he should be permitted to see them. On this promise George III did consent to the journey, and it did take place. But on coming to Kew the promise under which he had acted was not fulfilled; and the result—as might surely have been foreseen—was that same night a paroxysm of much increased severity.* * * *

"On the 4th of December the Parliament met in most anxious expectation. Mr. Pitt in the one house, and Lord Camden in the other, laid upon the table the Report of the Examination [of the physicians attending the King] before the Privy Council, and moved that it should be taken into consideration on the 8th. At the same time the Prime Minister gave notice that he should propose the appointment of a committee to search for precedents. Mr. Fox suggested a doubt (as Mr. Vyner had before him) whether it was quite consistent with the dignity of Parliament to make a report from the Privy Council the groundwork of their proceedings on a question of such extreme importance. Pitt declared that he was anxious to afford the most ample information, but pointed out that the Privy Council could take evidence upon oath, which a Committee of the Commons could not.

"Meanwhile it had become apparent to the small circle of the King's confidential servants, that, eminent as were the physicians in attendance, they had up to this time altogether failed in effecting any mitigation of his symptoms. Might not greater benefit follow from the treatment of some one who had more specially applied himself to the cure of mental maladies? Foremost among such practitioners in public fame stood Dr. Francis Willis, a clergyman and Rector of Wapping. By a somewhat unusual combination of duties he had, during twenty-eight years, kept an asylum for insane persons at his residence in Lincolnshire. His name was first brought forward by Mrs. Harcourt, the wife of one of the equerries, General, afterwards Earl, Harcourt. She drew up a paper stating her knowledge of his merits from his successful treatment of her mother. On the 28th of November, this paper being laid before the Prince, the Duke of York, the Chancellor, and Pitt, it was determined that Willis

* Vol. i., p. 398.

should be sent for. Accordingly he was summoned by express to Kew. He came accompanied by his two sons, one of whom, Dr. John Willis, was a professed physician; and on the 5th of December he had his first interview with the royal sufferer.

"From the first Dr. Willis formed a highly favourable opinion of the case. 'Had I been consulted in the first instance,' he frankly said, 'his Majesty's illness would have been of very short duration.' He adopted at once a different course of treatment. He laid aside all false pretences, all petty vexations, all unnecessary restraints. He thought that in this case no violence need be apprehended, and that no suspicion should be shown. The King had been denied a razor at his toilet, and a knife and fork at his table. These were at once restored to him, and in Dr. Willis's presence were freely used. The good effects of this altered treatment speedily appeared. As yet the delusions continued unabated, but far greater calmness and composure, as also better health, were attained.

"Under these circumstances both the Queen and Mr. Pitt were disposed to place great confidence in Dr. Willis. He was considered as mainly responsible for the conduct of the case. Taking up his residence with his sons in the palace at Kew, he had the constant care of the King's person, while the other physicians only paid their visits by rotation and at stated times.

On the 8th, at the next meeting of the House, Pitt adverted to the suggestions which had been made of appointing a Parliamentary Committee to examine the physicians, and declared himself willing to accede to it, observing that there was now a stronger reason for it than at the last debate, since some new physicians had been recently called in. Accordingly there was named, with general assent, a committee of twenty-one, comprising the most distinguished members from both sides. A similar motion was made and carried in the Lords, and each committee completed its examination in a single day. All the physicians, in their evidence, agreed that there were good hopes of the King's recovery, but that no probable period for it could be named. Yet the degree of these hopes was not the same in all, and here again party spirit crept in. Dr. Warren was closely connected with Fox and Fox's friends, and it was observed that his prognostics were far less sanguine than those of Dr. Willis. Thus the names of these two physicians became, as it were, watchwords on each side; the Ministerial party relying on the special experience of Willis, while the Opposition party might allege the high authority of Warren.

"On the 10th of December Mr. Pitt presented to the House the Report of these examinations, and observed that the King's present incapacity for business having now been ascertained by a committee of their own members, he should proceed according to his notice, and move that another committee be appointed to search for precedents.* . . . .

"The Regency Bill finally passed the House of Commons on the 12th of February. On the 17th and 18th the Peers discussed it in Committee. The third reading was close impending, and the Chancellor was ready to give immediately afterward the substitute for the Royal Assent.

"But a change was now at hand in the King's health. On the 2nd of February Miss Burney met the King by accident for the first time since the 5th of November. He was walking in Kew Gardens between the two Doctors Willis, and engaged her in conversation for a considerable time. She observed some wildness in his eyes, and a great deal of incoherence in his language. From the 6th the improvement grew rapid and decided. Dr. Willis, who was in daily communication with Mr. Pitt, declared himself clearly of opinion that after a short period no part of His Majesty's mental disorder would remain. For some time Dr. Warren was reluctant to acknowledge any real improvement

* Vol. ii., p. 8, et seq. 1789.

in the face of his gloomy predictions. But ere long he was overpowered by the force of facts. The public bulletin of the 12th of February mentioned 'progressive amendment,' and that of the 17th 'a state of convalescence.'

"Still Pitt and Lord Thurlow, feeling the magnitude of the point at issue, hesitated. But Dr. Willis was clear in his own opinion. He sought an interview with Lord Thurlow, and repeated that opinion in the strongest terms. As the story was afterwards told by one of his sons, Dr. Willis actually 'bullied' the Chancellor before he could make him stir in the matter.

"On the 19th, however, a Cabinet was held, and in consequence of the decision which was there adopted, the Chancellor, that same evening, rose in the House of Lords, and announcing the auspicious news, proposed that the Committee on the Regency Bill should be put off till the 23rd. 'And,' Miss Burney adds, 'this evening, for the first time, the King came upstairs to drink tea with the Queen and Princesses in the drawing-room. Huzza! huzza!'

"On that afternoon also Pitt wrote to his mother, after several weeks of silence, to give her the good news:—

"My dear Mother, "Thursday, Feb. 19, 1789."

"You will have seen for some days how constantly the news from Kew has been improving. The public account this morning is that the King continues advancing in recovery. The private one is that he is, to all appearance, perfectly well, and if it were the case of a private man, would be immediately declared so. It remains only to consider how far he can bear the impression of the state of public business; but, in consequence of these circumstances, the Bill will probably be postponed in the House of Lords to-day till Monday; and if the prospect is then confirmed, the plan of the Regency must probably be altered with a view to a very short interval indeed, or perhaps wholly laid aside. This intelligence will be welcome enough to excuse a short letter, and I could not resist the pleasure of communicating it, though not in a moment, as you may imagine, of much more leisure than even for some time past under different circumstances.

"Ever, my dear Mother, &c.,

"W. PITT."

"On the 20th the Chancellor went himself to Kew, and had an interview of an hour and a quarter with the King. He was with his Majesty again on the 22nd, and reported to Pitt that he never at any period had seen him more composed, collected, and distinct. 'On the 23rd,'—thus writes Mr. Grenville to his brother at Dublin Castle,—'the two Princes were at Kew, and saw the King in the Queen's apartment. She was present the whole time, a precaution for which, God knows, there was but too much reason. They kept him waiting a considerable time before they arrived, and after they left him drove immediately to Mrs. Armistead's, in Park Street, in hopes of finding Fox there, to give him an account of what had passed.'

"Later on the same day, the 23rd, the King wrote his first letter since his illness to Pitt; an excellent letter, as Wilberforce, to whom it was shown in confidence, calls it in his diary. On the morrow ensued the first interview between the Monarch and the Minister. Pitt, on his return from Kew, called upon Grenville, and gave him, in perfect unreserve, an account of what had passed. 'I was with the King,' he said, 'above an hour this morning. There was not the smallest trace or appearance of any disorder. His manner was unusually composed and dignified; but there was no other difference whatever from what I had been used to see. The King spoke of his disorder as of a thing past, and which had left no other impression on his mind than that of gratitude for his recovery, and a sense of what he owed to those who had stood by him. He spoke of these in such a manner as brought tears into his eyes; but with that degree of affection of mind there was not the least appearance of his disorder. After I had left his Majesty, I conversed with Willis, who told me that he now thought the King quite well; that he could not perceive the least trace remaining of his malady.'* * * *

"In the summer, the King, being advised to confirm his health by some sea-bathing, went to Weymouth, accompanied by the Queen and Princesses. His mode of life upon the coast is thus described:—'He usually rises at six, walks the Parade till eight, takes breakfast before ten, rides till three, dines at four, and resumes the promenade with the Queen and Princesses till late in the evening, provided the weather be fine. Sometimes the scene was varied by a sailing party on the sea, or an excursion inland; and their Majesties visited both Exeter and Plymouth before they returned to Windsor.'† * * *

1801.—"In the middle of February the King fell ill. His illness was at first no more than a feverish cold. On the 17th he saw Mr. Addington, and on the 18th he saw the Duke of Portland. With the latter he talked very calmly on the general aspect of State affairs. 'For myself,' said his Majesty, 'I am an old Whig; and I consider those statesmen who made barrier-treaties and conducted the ten last years of the Succession War the ablest we ever had.' The Duke only noticed as unusual that the King spoke in a loud tone of voice. But it is remarkable in this conversation that George III. discerned, what since his time has become much more apparent, how, not by any sudden change, but by the gradual progress of events, the Whig party has drifted away from its first position in the reign of Queen Anne, and come round to occupy the original ground of its opponents.

"The King's calmness in this interview did not long continue. A most grievous calamity was now impending over him from all the agitation and anxiety which he had just sustained. After an interval of twelve years his mind was once more deranged. The Duke of Portland was with him again on the 20th, and was then extremely alarmed. Next day, that is, on Saturday the 21st, the mental alienation was plainly manifested. On the Sunday Mr. Addington was for a short time admitted to his chamber, and afterwards reported to Mr. Pitt that he had found the King collected on some points, but wandering on others. Unhappily the symptoms, instead of diminishing, increased, and became at last not less acute than in 1788.

"It is said that one of the earliest symptoms which the King publicly showed of his mental affliction was in Chapel, and it may have been on this very Sunday. He repeated in a loud voice, and with extraordinary emphasis, as though referring to his own accession, in 1760, the well-known verse in the Morning Service: 'Forty years long was I grieved with this generation, and said: It is a people that do err in their hearts, for they have not known MY ways.'

"On the Monday the King was for many hours without speaking, and, it would seem, unconscious of what passed around him. Towards the evening he came to himself, and then said, 'I am better now, but I will remain true to the Church.' Thus at every intermission of his malady his mind at once reverted to the first cause of his distress. By an order of the Privy Council, public prayers were offered up for his Majesty's recovery, and the three Doctors Willis were summoned to his aid.

"On Tuesday, the 24th, however, Lord Loughborough, as still holding the Great Seal, thought himself justified by the public exigency in going to Buckingham Palace and obtaining the King's signature to a Commission for giving the Royal Assent to an Act of Parliament. That Act was for the repeal of the Brown Bread Bill, which, as I have elsewhere shown, was passed in haste at the close of the preceding year, and which had been found very mischievous in practice. There is no doubt that all parties now concurred in

* Vol. ii. p. 24 et seq. † Vol. ii. p. 37.

desiring its repeal, and that a delay of that repeal would have been injurious; yet even this consideration scarcely suffices to vindicate the course which, under such circumstances, the Chancellor pursued. On returning from the Palace, his Lordship said that when he had carried the Brown Bread Act to the King, his Majesty was in the possession of his understanding. But this was only his Lordship's public declaration. To Mr. Rose, as to a private friend, he owned that he had not seen the King at all. He had sent in the Commission to his Majesty by Dr. Willis, who brought it back signed, and told him that there would be no difficulty in obtaining the Royal signature to a dozen papers respecting which no detailed statements were necessary.

"During many days the King's malady did not abate. During many days he was unable to see his Ministers, either the late or the new, or even the Queen and the Princesses. Meanwhile the Government was in a most anomalous, nay, unprecedented state. Here was one Cabinet in progress of formation, and sanctioned by the King. Here was another Cabinet which had resigned; but still holding the seals of office, and alone competent to do any official act. Here was Mr. Addington, Prime Minister *de jure*. Here was Mr. Pitt Prime Minister *de facto*. It was only by the entire cordiality at this time between the two statesmen that confusion was avoided. They held several familiar conferences on the painful, but, as it seemed, unavoidable and close-impending question of a Regency.* * * *

"On the 2nd of March there was a crisis in the King's disorder. His Majesty was so ill that his life was almost despaired of; but having sunk into sleep, which continued for some hours, he awoke much refreshed, and from that time steadily mended. 'On the whole,' says Mr. Rose in his journal of the 3rd, 'the alteration for the better appeared to be most extraordinary. The King was thought so well that the Queen and the Princesses took an airing in their carriages. This account was brought to Mr. Pitt, while in bed, before eight o'clock, by Mr. Addington. . . . . Mr. Addington came again to Mr. Pitt late in the day, when I was with him, and said the accounts from the Queen's House continued as favourable as possible.'

"During the next two days the King's health continued, though slowly, to improve. Nevertheless, on the 5th Pitt felt it necessary to consider seriously with his Treasury intimates how far it would be possible to prolong the *Interregnum*. It was absolutely requisite to obtain, without much further delay, the Royal sanction to the foreign despatches, and the Royal assent to the Parliamentary Bills. Pitt came to the conclusion that unless his Majesty should be quite well before the 12th, that was the latest day to which he could defer an examination of the physicians either before the Privy Council or the House of Commons. In that case a Regency Bill might be brought in on the 14th, and might pass by the 23rd. This was on the supposition that it would be unopposed. And Mr. Pitt thought that it would not be safe to defer the inquiry of the physicians even till the 12th, unless it could be ascertained that no delay would be created.

"All questions of Regency, however, were set at rest by the King's convalescence. It is remarkable that the first favourable change was due to Mr. Addington, not, indeed, in his political, but rather in his filial capacity. He remembered to have heard from his father, the eminent physician, that a pillow filled with hops would sometimes induce sleep when all other remedies had failed; and the experiment, being tried upon the King, was attended with complete success. Some persons have supposed that a rumour of this fortunate prescription gave rise to the nickname of 'The Doctor,' which, some months later, was almost universally applied to Mr. Addington; but I doubt whether

* Vol. iii. p. 292 et seq.

the report was ever so prevalent as to produce that popular taunt, which was only, I conceive, a reminiscence of his father's profession.

"On Friday, the 6th of March, the King, though much reduced in strength, was clear and calm in mind. He sat for some time with the Queen and the Princesses. He desired Dr. Thomas Willis to write an account of his convalescence to Mr. Addington, to Lord Eldon, and to Mr. Pitt. With respect to Mr. Pitt, his Majesty used the following words:—'Tell him I am now quite well—quite recovered from my illness; but what has *he* not to answer for who is the cause of my having been ill at all?' [Referring to Mr. Pitt's conduct on the Catholic Question.]

"Pitt was deeply affected. It had given to him and to his colleagues, who were retiring from the Cabinet, most heartfelt pain to find that their conscientious course of duty had been the means of bringing upon their Royal Master this heavy and unforeseen affliction. Lord Malmesbury has an entry as follows in his journal, under the date of the 25th of February:—'Lord Spencer very much hurt at what has passed, and feeling a great deal for the share he has had in it; and Pitt, though too haughty to confess it, feels also a great deal.'* * * *

1804.—"On the 12th or 13th of February, Lord Malmesbury states, the King (after having taken cold by remaining in wet clothes longer than should be) had symptoms of the gout. He could not attend on the Queen's birthday, though he appeared in the evening at an assembly at the Queen's House; he was too lame to walk without a cane; and his manner struck me as so unusual and incoherent, that I could not help remarking it to Lord Pelham, who, the next day (for I went away early) told me that he had, in consequence of my remark, attended to it, and that it was too plain the King was beginning to be unwell. Lord Pelham, who played that evening with the Queen, added that her anxiety was manifest, since she never kept her eyes off the King during the whole time the party lasted.

"The King was at first attended only by his household physician. He had conceived a strong dislike to the Doctors Willis, from the treatment which they had found requisite in his malady three years before—a feeling very frequent with persons in that afflicted condition. At his urgent request, as his illness increased, another physician, Dr. Symonds, was called in. For two days his Majesty's life was in danger, and for at least a week the derangement of his mind was complete. By degrees he began to rally, but more slowly and with a greater tendency to relapse than either in 1789 or 1801."† * * *

In May the mental state of the King continued to be a matter of anxiety. Mr. Pitt noticed some increase of agitation on the 10th and later in the month.

"The Duke [of Portland] told Lord Malmesbury that he had little doubt of the King doing well. Quiet would set him right, and nothing else. Not quite so sanguine was Mrs. Harcourt, who came to see Lord Malmesbury the next day. She said that the King was apparently quite well, when speaking to his ministers, or to those who kept him a little in awe, but that to his family and dependents his language was incoherent and harsh, quite unlike his usual character. He had made capricious changes everywhere, from the Lord Chamberlain to the grooms and footmen. He had turned away the Queen's favourite coachman, made footmen grooms, and *vice-versâ;* and, what was still worse, because more notorious, had removed Lords of the Bedchamber without a shadow of reason. All this, said Mrs. Harcourt, afflicts the Royal Family beyond measure. The Princesses are quite sinking under it.

"The 27th of May, on which Lord Malmesbury wrote these last entries in his journal, supplied another proof that even a mere trifle might still throw the King's mind from off its balance. Going down to Windsor on the 26th, his

* Vol. iii. p. 302. † Vol. iv. p. 119.

carriage was followed some way, and loudly cheered, by a party of Eton boys. This had such an effect upon his Majesty, that when he met a different party of the boys next morning, he said to them, 'I have always been partial to your school. I have now the additional motive of gratitude for being so. In future I shall be an anti-Westminster.'

"Yet Mr. Rose, from whom we derive this story, declares that when on the 5th of June he attended a Council to kiss the King's hand, on his appointment —which was the first time he had seen his Majesty since his recovery—'the King spoke to me for about ten minutes, and I never saw him more entirely composed and collected; if anything, less hurried in his manner than usual.'* * * * *

1805, July 1.—"His Majesty was now suffering under a grievous calamity, the most grievous in this world perhaps, or second only to the mental aberrations which he had also undergone. He was beginning to lose his sight. One eye was almost entirely darkened, and the other grew less and less clear. There was a hope, but as it proved not well founded, that the advance of the cataract would leave scope hereafter for a successful operation. Meanwhile the King bore his distress with the greatest fortitude and resignation."† * * *

In a letter to Mr. Pitt, dated from Weymouth, August 26, 1804, the King says:—

"As to Mr. Pitt's inquiries as to the King's health, it is perfectly good, and the quiet of the place and salubrity of the air must daily increase his strength. By the advice of Sir Francis Milman who is here, the King will bathe in the tepid bath, in lieu of going into the open sea. His Majesty feels this a sacrifice, but will religiously stick to this advice, but does not admire the reasoning, as it is grounded on sixty-six being too far advanced in life for that remedy proving efficacious."‡

In September, Mr. Pitt, in the hope "that the experience of the past year might make the King less adverse to his counsels for an extended basis of administration," had an interview, of three hours' duration, with his Majesty, at Weymouth. He "urged anew, but quite in vain, all the arguments he could, stopping short only at the point when he feared lest he might disturb the health or the mind of his Royal Master."§

A day or two prior to this interview, the King addressed a brief note to Mr. Pitt, which terminated with the following remark:—"His Majesty's sight will not allow him to add more, as though he gains some ground, he can neither read what is written to him nor what he writes."

Towards the end of October, 1810, the King's mind became again disordered, and from this attack he never recovered, continuing in a state of mental incapacity until his death, which occurred at Windsor on the 29th January, 1820, in the eighty-second year of his age. "All history," touchingly says the author of the "Four Georges," "presents no sadder figure than that of the old man, blind and deprived of reason, wandering through the rooms of his palace, addressing imaginary parliaments, reviewing fancied troops, holding ghostly courts. I have seen his picture as it was taken at this time, hanging in the apartment of his daughter, the Landgravine of Hesse Hombourg—amidst books and Windsor furniture, and a hundred fond reminiscences of her English home. The poor old father is represented in a purple gown, his snowy beard falling over his breast—the star of his famous Order still idly shining on it. He was not only sightless: he became utterly deaf. All light, all reason, all sound of human voices, all the pleasures of this world of God, were taken from him. Some slight lucid moments he had, in one of which the Queen, desiring to see

---

* Vol. iv. p. 194. † Vol. iv. p.

‡ Vol. iv. *Appendix*, p. xviii. § Vol. iv. p. 334.

him, entered the room, and found him singing a hymn, and accompanying himself on the harpsichord. When he had finished, he knelt down and prayed aloud for her, and then for his family, and then for the nation, concluding with a prayer for himself, that it might please God to avert his heavy calamity from him, but if not, to give him resignation to submit. He then burst into tears, and his reason again fled."

---

### THE "ÆSTHETICS OF SUICIDE."

